# Current epidemiological and etiological characteristics and treatment of seizures or epilepsy in patients with HIV infection

**DOI:** 10.1186/s42494-020-00028-8

**Published:** 2020-10-21

**Authors:** Changhao Yu, Dong Zhou, Weijia Jiang, Jie Mu

**Affiliations:** 1 West China Hospital of Stomatology, Sichuan university, 14# Third Section, Renmin Nan Road, Chengdu, 610041 China; 2 Neurology Department, West China Hospital of Sichuan University, 14# Third Section, Renmin Nan Road, Chengdu, 610041 China; 3 West China Medical Publishers, West China Hospital of Sichuan University, 14# Third Section, Renmin Nan Road, Chengdu, 610041 China

**Keywords:** HIV, Seizure, Epilepsy, Opportunistic infection, Antiepileptic drug

## Abstract

Seizures or epilepsy is one of the common serious complications in patients with advanced human immunodeficiency virus (HIV) infection or diagnosed with immune deficiency syndrome, with higher incidence and prevalence than in the general population. Generalized seizures are the most common type in the patients. Opportunistic infections are a stereotypical predisposing factor for seizures in HIV patients, but a variety of pathogenic factors can also be found in these patients, such as metabolic perturbation and drug-drug interactions. The diagnostic criteria for seizures in these patients are the same as those in the general population. As HIV patients with seizures need to take both antivirals and antiepileptic drugs, the risk of drug-drug interactions is greatly increased, and the side effects of drugs may also become more prominent. At present, most experience in antiepileptic drug usage has come from the general population, and there is still a lack of guidance of antiepileptic drug use in special groups such as the HIV-infected people. Unlike the old-generation drugs that involve metabolisms through CYP450, the first-line antiepileptic drugs usually bypass CYP450, thus having less drug-drug interactions. In this review, we summarize the recent research progress on the above-mentioned widely discussed topics and make a prospect on future research direction.

## Background

Patients living with advanced human immunodeficiency virus (HIV) infection or diagnosed with acquired immune deficiency syndrome (AIDS) usually have complications related to the central nervous system (CNS), which may include psychiatric disorders, encephalitis, cerebrovascular diseases, etc. [1–3]. Seizures or epilepsy is a serious type of complication. Patients with witnessed seizures need long-term periodic medication control, making it a challenge for clinical drug management. In pediatric patients living with HIV, seizures or epilepsy may be concurrent with brain development delay or neuron-cognitive impairment [4]. Besides, HIV-seropositive patients with documented seizures are at a higher risk of developing other CNS syndromes, compared with the HIV-negative patients [5]. Among these patients, opportunistic infection (OI) as a result of deep immunosuppression is the most frequent cause; however, there is a considerable proportion of patients with no predominant causes, suffered from HIV encephalopathy (HIVE). Another part of patients has seizure relapses due to the interactions between antiepileptic drugs (AEDs) and antiretroviral drugs (ARVs), which makes seizure improperly controlled.

In addition, the decades of development on treatment options clinically proven to be effective and widely accepted for seizures and epilepsy arise from populations sufferring only from seizures or epilepsy. Targeted research or treatment plans for HIV-seropositive patients with documented seizures are needed. Epidemiological research has advanced our understanding of the distribution characteristics of seizures or epilepsy in the HIV+ population. This may help formulate new medication protocols for HIV+ patients with epilepsy.

This review updates research information from the epidemiological characteristics of seizures and epilepsy in the HIV-seropositive population to the causes and then the therapeutic protocols. We also review the survival status and medication methods of HIV+ patients with documented seizures or epilepsy, in order to provide clinical decision-makers with the latest progress to refer to.

## Epidemiological characteristics

### The prevalence and incidence of seizures in the HIV+ population

The incidence and prevalence of seizures are higher in the HIV-infected population than in the general population [6–9]. As demonstrated in previous research, the incidence and prevalence of seizures in the general population range 0.4–1.0% [10] and 0.05% [11], respectively, while those in the HIV+ patients (including the patients with documented seizures prior to HIV infection and patients with newly-onset seizures [NOS] after the infection of HIV) are 1.8–19.8% and 2–19.8%, respectively [7, 8]. These two statistical indicators varies among different countries/regions or longitudinally in time. Studies from centers in Africa peculiarly tended to report a higher prevalence or incidence, while the developed countries usually reported lower incidence. A retrospective study from Ireland showed that the incidence of seizures is estimated to be doubled in the HIV+ population than in the local general population (with the incidence of seizures is 2.4%) [7]. However, after a 5-year retrospective study, Kim et al. from Korea found the incidence of seizures in HIV+ patients was 3.0%, compared with 0.24% in the general population [12]. Samia et al. from South Africa reported both prevalence and incidence of seizures in the included HIV-infected children as 7.6% [13]. Another study from South Africa reported a 23% rate of history of seizure in 227 involved HIV+ children [14]. This situation may be due to the pandemic of AIDS in Africa, where many children were afflicted through the maternal-neonatal transmission. What’s worth noticing is that reports from India also revealed a relatively higher distribution of seizures in HIV+ patients. Sinha et al. reported an overall 20% proportion of NOS in a local hospital [15].

Further, studies carried out in around 1990 reported a higher incidence. Wong et al. and Holtzman et al. reported the incidence of NOS in New York to be 11 and 18%, respectively [16, 17]. Before the invention of highly active antiretroviral therapy in1996, patients were more likely to suffer from complications caused by increased virus load.

Gender does not seem to influence the prevalence or incidence. Studies have shown that the sex ratio was similar between the enrolled HIV+ population and patients with seizures [7, 13, 18, 19]. This suggests that, in some studies where the NOS occurrence was 3–10 times higher in male patients than in female patients, the imbalance in the sex ratio of recruited HIV+ patients might explain the different occurrence [6, 12, 16]. However, whether gender plays a role in the susceptibility still needs more rigorous research to prove.

At present, there is no obvious evidence that the age factor could promote or reduce the occurrence of seizures in HIV+ patients to a certain extent. Although children are more vulnerable to CNS infections than adults, affected by the heterogeneity between different studies, it cannot be asserted that the HIV+ children are more prone to seizures than adults. Notably, studies in different regions showed that the mean age of patients at NOS occurrence was 30–40 years old [7, 19, 20].

As for the prevalence or incidence of epilepsy in the HIV+ population, most studies only reported the prevalence or incidence of seizures in detail, and only a few reported recurrent rates and followed up to the diagnosis of epilepsy. From the three studies reported so far, the proportion of HIV+ patients who had seizures and were subsequently diagnosed with epilepsy was very high (37/38, 21/27, and 32/52) [6, 13, 14]. It can be considered that the epidemiological characteristics of epilepsy in HIV+ patients may be similar to seizures.

### The predilection types and occasions of seizures or epilepsy

Seizures are reported to emerge at different stages of HIV infection, and are manifested as the initial clinical symptom in some patients after HIV infection. For example, some patients are hospitalized for seizure occurrence and screened to be HIV-seropositive afterward. Under most circumstances, patients are at the advanced stage of infection, when the OI becomes a common event, from which seizures arise. And it seems that the more advanced stage of HIV infection, the greater the likelihood of seizures to occur. Indeed, many patients inflicted seizures after the diagnosis of AIDS [16]. Satishchandra et al. discovered that seizures were the presenting manifestation in one of five cases in their series [21]. Intriguingly, the majority of seizures in HIV+ patients in their study were still present at the stage of advanced infection. From the statistical data, seizures are unlikely to be conceived of as an exceptional complication of advanced HIV infection, but they have an adverse impact on the quality of life.

According to the epilepsy definition by International League against Epilepsy (ILAE) in 2014 [22] and seizures classification in 2017 [23], most seizures in HIV-infected patients are generalized. The proportion of generalized seizures in patients with NOS ranged from 35.3% [12] to 67.3% [14].

The epilepsy type could not be demonstrated in HIV-infected patients because of a lack of studies. Some studies have reported the existence of status epilepticus (SE) in their HIV-infected participants, with a proportion from 0.175 to 4.34% [7, 12, 14, 20], while in a meta-analysis published in 2017, status epilepticus had a prevalence of 12.6/100,000 among general populations [24]. This suggests that HIV may be an underlying risk factor for status epilepticus. However, since the etiological evidence of patients with status epilepticus has not been stated, the possibility of other causes cannot be ruled out. To figure out the relationship between HIV infection and status epilepticus, further research is needed.

## Etiological characteristics

On the whole, the etiology of seizures in HIV-infected patients comes from HIV itself and other causes induced by HIV infection. The drug-drug interactions are also responsible for the recurrence of seizures, and they will be discussed in detail in the following therapeutic sections. Even dating back to 30 years ago, the OIs were also reported as the most dominating cause of seizures in HIV-infected patients [17]. HIV+ patients are more susceptible to pathogenic microbes such as toxoplasmosis, cryptococcus species, tuberculosis, and John Cunningham virus (JCV), four most common pathogens for OI, due to the immunosuppression. Typically, it is when the CD4 count is lower than 100 cells/μL that these pathogens cause encephalitis [25]. Nevertheless, tuberculosis is an exception that can cause tuberculous meningitis (TBM) at any level of CD4 count as long as there is immune suppression [26]. Following the widely-adopted highly active antiretroviral treatment (HAART), both the incidence and mortality of these OIs have declined. According to a most updated meta-analysis, OIs account for nearly 50% of the causative factors in approximately 68% of HIV+ patients experiencing NOS with conclusive causes, with toxoplasmosis diagnosed in the majority (21%), followed by progressive multifocal leukoencephalopathy (PML) and cryptococcal meningitis (14% for each) [9]. This paper focuses on several regular infectious pathogens and non-infectious agents.

### Toxoplasmosis

Infection with *Toxoplasma gondii*, an obligate intracellular parasite, is the cause of toxoplasmosis. Domestic cats are the only known definitive hosts of *T. gondii* and often lead to the indirect infection of the parasite in humans through food contamination with spore-forming oocysts. *T. gondii* is one of the most common infectious agents responsible for newly-onset seizures in HIV seropositive patients [18, 27, 28]. They are often afflicted with focal seizures caused by mass lesions. CT or MRI scan often shows single or multiple nodular lesions. However, even if no hypointense lesions could be seen, some silent lesions still revealed themselves at autopsy. CSF biochemical examination is a more reliable choice for diagnosis than serologic assays, and a study reported a sensitivity of 75% with the use of B1 gene nested PCR [29]. However, the lumbar puncture cannot be administered to patients with increased intracranial pressure due to the high risk of cerebral hernia; in this circumstance, how to diagnose and when to decide to start treatment usually rely on empirical experience. The routine protocol for HIV+ patients is a long-term consecutive inhibition therapy until continuous antiretroviral therapy raises the CD4 count over 200 cells/μL. And a standard therapeutic plan is the co-administration of pyrimethamine, sulfadiazine, and folinic acid. Besides, a prospective study from Cameroon concluded that newly-onset seizures in these patients have a benign clinical course and favorable prognosis, thus not requiring AEDs [30].

### Cryptococcal meningitis

It is convinced that cryptococcal meningitis can be caused by the spread of fungal pulmonary infections. It is the second most common HIV-associated OI among adults in the sub-Saharan Africa [31], and the global prevalence of cryptococcal infection has been estimated to be 6% in HIV+ patients [32]. According to a prospective study, in 28% of the HIV-infected cryptococcal meningitis participants, the generalized seizures were regularly witnessed [19]. CSF analysis is a piece of important evidence for diagnosis. The opening pressure often appears to be greater than 25 cmH_2_O [33]. CSF cultures are definitive standards, but it may take weeks to reveal a positive result. The India Ink assay can assist diagnosis, but a negative result cannot deny the possibilities of positive results. Results of imageology may be nonspecific due to the nonmeningitic neurologic complications of HIV, such as atrophy in HIVE patients. The prognosis of such patients is correlated with the CSF opening pressure. Patients with seizures tended to have higher opening pressure, which has an inverse relationship with the occurrences of seizure attacks. For the treatment of HIV+ patients, an adequate treatment course at the beginning is recommended. The administration of amphotericin B should not be interrupted until the CSF culture turns negative. And a consistent maintenance therapy using fluconazole should follow the initial therapy as far as CD4 count is over 100 cells/μL [34]. In patients with NOS, cautions should be given to AED applications due to the potential drug-drug interactions and the latent impairment in renal clearance [19]. Therefore, it is more practicable to screen asymptomatic *cryptococci* infection among HIV-seropositive patients, thus facilitating prompt prevention and treatment.

### Tuberculous meningitis (TBM)

The seizure is usually secondary to TBM, with previous studies reporting an incidence of 11.3–39.1% [35, 36] and a high possibility to reoccur. The co-infection with HIV is a strong risk factor for progression into active tuberculosis, seizures may occur not only during the active phase. TBM can present with seizures in 10–16.3% of patients [37, 38]. The type of seizures can be generalized, or focal with or without secondary generalized seizures. The contrast-enhanced MRI is more helpful than CT in confirming seizures in patients with TBM [39]. The outcomes of background slowing and epileptiform discharges in EEG and hyponatremia in CSF laboratory tests suggest a high risk of seizure occurrence [40, 41]. Patients with non-single seizures often have a poor prognosis. Because some AEDs and antituberculosis drugs have drug interactions [42], it is controversial whether to administer AED in patients with single seizure. Under this condition, cortical involvement and epileptiform discharges are important reference indicators [36].

### Progressive multifocal leukoencephalopathy (PML)

PML is a demyelinating disease of the CNS characterized by extensive lesions ascribed to the infection of oligodendrocytes by the JC virus. It occurs almost exclusively in patients with immunosuppression, and its prevalence in HIV-infected individuals is around 4–5% [43, 44]. A considerable amount of studies have described PML as a crucial cause of seizures in such a population, and the incidence of seizures was found to be 18% [4, 12, 45, 46]. The type of seizures can also be focal or generalized. PCR of CSF is a reliable tool for diagnosis. Neuroimaging often shows single or multiple fusion lesions without mass effect. Nevertheless, patients presenting with seizures are inclined to have demyelinating lesions in the immediate vicinity of the cortex. Usually, treatments have satisfying effects on seizures.

### HIV-associated meningitis (HIVE)

No noticeable pathological factors could be found among a considerable percentage of HIV+ patients [6, 13]. More and more researchers tend to presume that HIVE is a consequence of the direct impact of HIV on the CNS [21, 47]. An early form of aseptic, HIVE develops within days to weeks after HIV infection [48]. Some researchers found microglial nodules and multinucleated giant cells in the brains of patients at autopsy and believed that this may be the evidence that HIV induces central neurological conditions [16].

### Other non-infectious causes

Patients infected with HIV are prone to serum electrolyte distributions, such as hyponatremia, hypomagnesemia, and hypocalcemia. It is well known that hypomagnesemia can induce seizures. And a cross-sectional survey showed that hyponatremia is also a risk factor for seizures with decreased serum sodium levels, resulting in an ascending risk [49]. Although electrolyte disturbances are common among HIV+ patients with seizures, only one study clearly stated that two patients had seizures as a consequence of hyponatremia and hypomagnesemia, respectively [20]. Hyponatremia is likely to play an important role in inducing seizures. As most patients may have more conspicuous etiology presented (such as OI), the electrolyte disturbances are easily to be neglected.

As we all know, HIV and HIV-related factors are high risk factors for stroke. It has been reported that HIV infection increases the risks of both ischemic and hemorrhagic stroke and CNS OIs also significantly increase the risk of stroke [50]. Stroke can lead to acute seizures or post-stroke epilepsy. But few reports have addressed HIV+ patients who developed seizures due to stroke. This may be related to the age distribution of the patients included in cohorts, or may be masked by a more conspicuous cause.

## The diagnosis

The diagnosis of epileptic seizures and epilepsy among HIV+ patients is still based on the distinctive clinical manifestations accounted by witnesses, combined with EEG and features of radiological examinations. The clinical manifestations consist of loss of consciousness (seizures, transients, stereotypes, or repetitive), limb rigidity, locked jaw, gaze, biting the tongue, mouth foaming, and hypermobility.

Currently, the diagnosis of epilepsy follows the diagnostic criteria established by ILAE in 2014 and the classification system by ILAE in 2017 [22, 23], as follows:

1. at least two unprovoked (or reflex) seizures occurring > 24 h apart;
2. one unprovoked (or reflex) seizure and a probability of further seizures similar to the general recurrence risk (at least 60%) after two unprovoked seizures, occurring over the next 10 years;
3. diagnosis of an epilepsy syndrome.

## Therapeutic tool

The general medical treatment goal of seizures is to control acute symptoms and prevent a recurrence. Due to the immunosuppression effect that HIV has on the host, and the frequent combined symptoms such as wasting syndrome in patients, the prevention of seizure and treatment of epilepsy often become a long-term process. Therefore, this population is routinely treated medically. Surgical therapies or vagus nerve stimulation should be considered if medication-resistant epilepsy exists and is estimated to be effective. Surgical treatment is beyond the scope of this article. The following will discuss the AED usage in HIV+ patients.

### Basic principles in AED selection

As is specified by the National Institute for Health and Care Excellence [51], AED selection should be rationalized depending on the type of seizures and the different phases and duration of seizures. If patients are in the acute seizure attack phase, the first aim is to control recurrence and reduce complications. Intravenous injection of benzodiazepines (such as diazepam) should be given. Assessment of a NOS is important. If it lasts less than 20 min, status epilepticus is beyond consideration, so oral AEDs and treatment of the cause are recommended. Unless there is a risk of developing status epilepticus, treatment for the first unprovoked seizure is not advised [52]. Any seizure that becomes prolonged or recurrent seizures without recovering consciousness between the seizures can be considered to have progressed to status epilepticus. Benzodiazepines (such as lorazepam, diazepam, or midazolam intravenous injection) are still the first-line drugs for initial status epilepticus [53].

When a NOS occurs, the decision on how long to take AED treatment is largely related to the risk of recurrence. Patients with a 60% risk of recurrence as abnormal EEG or brain lesions should consider the regular course of treatment with AEDs. Because of the high recurrence rate of seizure in HIV-infected patients, some scholars have suggested that long-term medication plans should be customized once NOS appear [21]. However, there are also objections, suggesting short-term AED treatment, on the grounds of avoiding potential side effects of drug and the AED-ARV interactions [7].

### Drug-drug interactions between AEDs and ARVs

Older-generation AEDs often have a drug interaction with ARV, especially in the case of joint use of enzyme-inducing AEDs (carbamazepine, phenobarbital, phenytoin) and nonnucleoside reverse transcriptase inhibitor (NNRTI or efavirenz for example) and/or protease inhibitor (PI). This results in either an increase in serum levels of AED or ARV, which will increase the risk of potential drug toxicology; or a decrease in serum level, which will lead to virologic failure or seizure recurrence. A study has shown that the combined use of carbamazepine and efavirenz reduces the serum concentration of efavirenz [54]. Another study showed that the half-life of nevirapine in patients treated with phenytoin sodium was significantly reduced [55]. This is because this generation of AED is mainly oxidized and metabolized by the CYP450 enzyme system, but efavirenz is both an inhibitor and an inducer of CYP3A4. Some AEDs are also partially (valproic acid, phenobarbital, or oxcarbazepine) or completely (lamotrigine) eliminated through conjugation. As PIs are becoming well-received among HIV+ patients, these two classes of drugs also interact mutually. It has been reported that when combined, lopinavir/ritonavir and atazanavir/ritonavir both lead to a significant decrease in the bioavailability of lamotrigine [56]. However, efavirenz is unlikely to interfere with AEDs and make seizure easier to recur; AEDs that are mainly dependent on conjugation degradation generally do not affect ARVs and result in virologic failure, at the regular dosage of course [42].

### First-line drugs in clinical practice

As for the first-choice AEDs, like levetiracetam, lacosamide, gabapentin, and pregabalin, a common feature of them is that they are neither metabolized via the CYP450 enzyme system nor rely on conjugation to eliminate. The main route of clearance of these AEDs is renal excretion. Though having no significant drug interactions with ARVs [57, 58], they still face restrictions in application. Levetiracetam, which is reported to have the least AED-ARV interactions, often causes intolerable neuropsychiatric side effects [59, 60]. And deliberate assessment of the tolerance of patients with impaired renal function should be performed when gabapentin or pregabalin is administered.

When conditions are not qualified to use the recommended first-line drugs, valproate can be used instead. In the human body, more than 50% of valproate is oxidized by CYP450, and the remaining is eliminated by conjugation. But so far, there is no clear evidence that its combined use with ARV can cause virologic failure. As valproate has dual inhibiting activities on CYP and uridine glucuronyl transferases (UGT), its combined use with ARV (zidovudine) or PI (lopinavir/ritonavir) has been reported to increase valproate serum levels [61]. In vitro experiments have shown that valproate stimulates HIV replication [62], but this conclusion has not been confirmed in vivo. On the contrary, a study reported a significant reduction of latent HIV in resting T cells in 3 of 4 patients when valproate was combined with HAART [63]. Therefore, valproate is a promising option when first-line AEDs are not available.

How to prevent seizure recurrence in HIV+ patients can be viewed from another perspective. Since OIs account for most NOSs, managing to reduce the viral load and increase CD4 count are also effective ways to decrease the risk of seizure recurrence. A cohort study found that the incidence decreased by about 1% after ART administration [64]. Another strictly controlled retrospective case-control study showed that earlier implementation of combination antiretroviral therapy (cART) was likely to prevent HIV-infected children from eventually developing epilepsy [65]. A study even suggested that it was safe to apply more than one AEDs in HIV+ patients currently treated by cART, as it was helpful to restore the host’s immunity, as long as its cumulative dosage was under an adaptable level [66].

Based on the above discussion, it is prospective to have a new therapeutic conception as co-administered AED and ARV to offset mutual drawbacks without an ascending drug toxicology risk.

### Medication for pediatric patients

Compared to adults, children with HIV infection are more sensitive to medication dose and are more vulnerable to side effects [67, 68]. Children are at a stage of growth and development, and are susceptible to the developmental delay and even cognitive developmental delay due to the adverse effects of HIV on the brain. Therefore, therapeutic protocols in children should keep balance between the efficacy and side effects of AEDs and ARVs. At present, there is no worldwide guideline on the optimal medication for children, and the Department of Health in South Africa recommends sodium valproate as a first-line drug [13]. But what is worth noting is that in some poor-resource regions, the old generation of AEDs is the only choice to reach.

As mentioned above, performing cART at the correct time can reduce the risk of seizures, which is of vital importance for prognosis in HIV-infected children. As the immune system of children is not well-developed, HIV infection at this stage may deteriorate the immune system and result in seizures. Studies showed that there was no significant difference in the percentage of cognitive impairment between children with early and delayed administration of cART [65, 69]. Instead, children who deferred the treatment are prone to developmental delays and even cognition obstacles due to HIVE or other CNS diseases complicated by HIV [70]. This suggests that the benefits of early implementation of cART may outweigh the more cumulative drug toxicity from longer use. And given the special situation in Africa, some scholars have suggested that ART should be carried out once children have any CNS complications [71, 72].

## Suggestions on future research on seizures in HIV-infected populations

### Future epidemiological research

Over the decades, enormous amounts of research have been carried out on the prevalence and incidence of seizure and epilepsy among HIV-infected populations. However, it is a challenging task to precisely describe the distribution of seizures and epilepsy among the HIV+ population, due to the following four reasons.

1. Up to now, nearly all the relevant studies are retrospective instead of being prospective, which may inappropriately include the cases who had an undocumented seizure before HIV infection, thereby exaggerating the incidence of NOS.
2. Most of these studies were performed in selected cases from one or two local medical establishments, and large-scale cross-sectional investigation is imperatively needed. The available findings may have geographic limitations and lack broader geographical representation. For example, the conclusions drawn from neurological medical centers may show a higher prevalence than from the general hospitals, because more refractory patients are referred to the former. Results of local hospitals may underestimate the prevalence as it is difficult to guarantee the representativeness of the sample patients for the whole HIV-infected population.
3. Many of the previous studies focused on the incidence or prevalence of seizures but neglected the development of epilepsy. In the past 20 years, the diagnostic and classification criteria of epilepsy and seizures have changed several times. For some quite early studies, it is hard to determine whether the diagnosis was based on internationally recognized diagnostic standards and whether the diagnosis at that time could be classified into the corresponding revised diagnostic entries nowadays.
4. In some studies reporting epilepsy prevalence and incidence, the diagnostic standards was not clarified. As these studies used a retrospective setting, the authenticity of the data may be questionable.

The frequent types of epilepsy and seizures are also controversial. It is well known that the seizures caused by acute CNS infections secondary to HIV-immunological suppression are mostly focal, but what’s the paradox is that generalized seizures dominate in a large number of reports [6, 9, 13, 18, 21, 72]. This may be explained by the possibility that the secondary generalized seizures were mistakenly regarded as generalized seizures, that is, the initial focal symptoms were not witnessed. This contradiction also reflects a pervasive retrospective bias that cannot be ignored in these retrospective studies.

Suggestion on this section: we appeal for more prospective studies in the future to eliminate the retrospective bias. Before describing the occurrence of seizures or epilepsy, a statement of the diagnostic procedure (or obedience of the acknowledged diagnostic criteria) should be delivered. And it is advisable to distinguish the chronological order between NOS and HIV infection when making medical records. However, when the time point of HIV infection cannot be determined, the ART start time is recommended as an alternative.

### Future etiological studies

Although HIVE has been largely reported in numerous studies, it remains doubtful whether HIVE is as frequent as reported. According to the Centers for Disease Control and Prevention, HIVE cannot be diagnosed unless the patient meets at least one of its criteria for ≥2 months [73]. Only few studies stated that the diagnosis of HIVE strictly followed the standard [14]. Most literature merely attributed HIVE to seizures in patients with no obvious etiology. Considering that these studies were mainly retrospective, the incidence of HIVE may have been exaggerated.

Suggestion on this section: the majority of HIV+ patients with seizures are often affected by several etiological factors, and these risk factors may reinforce each other. It is recommended that researchers carefully check for potential causes that could be missed. For prospective studies, a well-recognized diagnostic criterion should be used. For retrospective studies, HIV infection cannot be blindly attributed to patients with no obvious pathogenic factors.

### Future studies on the treatment plan

The current mainstream AED guidelines are based on the results of clinical trials in the general population, rather than being specific to the HIV+ population. Seizures in HIV+ patients are mostly a consequence of OIs. It is common to administer multiple drugs for different pathological conditions at the same time, considering the patients’ antidote competence, drug interactions, toxicology, and side effects. Sometimes even giving up medication for a certain etiological agent should be considered.

Suggestion on this section: we call for cohort studies that specifically include HIV+ epilepsy patients. Choosing drugs with different metabolic pathways may decrease the likelihood of drug-drug interactions.

## Conclusion

In summary, this paper discuss the clinical characteristics and treatment protocols of HIV+ patients with seizures or epilepsy. As a frequent serious complication of HIV infection often caused by OIs, seizures have a prevalence of 2–19.8% and incidence of 1.8–19.8% among the HIV-infected population, and mostly appeared in a generalized type. The contemporary medication therapy for seizures or epilepsy is well-developed, but largely based on the general population. Future therapeutic studies in HIV+ patients with seizures are urgently desired.

## Data Availability

Not applicable.

## Abbreviations

AED: Antiepileptic drug
AIDS: Acquired immune deficiency syndrome
ART: Antiretroviral therapy
ARV: Antiretroviral drug
cART: Combination antiretroviral therapy
CNS: Central nervous system
CSF: Cerebrospinal fluid
CT: Computed tomography
CYP: Cytopigment
EEG: Electroencephalogram
HAART: Highly active antiretroviral therapy
HIV: Human immunodeficiency virus
HIVE: HIV encephalopathy
ILAE: International league against epilepsy
MRI: Magnetic resonance imaging
NNRTI: Non-nuceloside reverse transcriptase inhibitor
NOS: New-onset seizure
OI: Opportunistic infection
PCR: Polymerase chain reaction
PI: Protease inhibitor
PML: Progressive multifocal leukoencephalopathy
SE: Status epilepticus
TB: Tuberculosis
TBM: Tuberculous meningitis
UGT: Glucuronyl transferases
